# TRAIL-mediated killing of acute lymphoblastic leukemia by plasmacytoid dendritic cell-activated natural killer cells

**DOI:** 10.18632/oncotarget.4984

**Published:** 2015-07-22

**Authors:** Martin Lelaidier, Yildian Dìaz-Rodriguez, Martine Cordeau, Paulo Cordeiro, Elie Haddad, Sabine Herblot, Michel Duval

**Affiliations:** ^1^ Groupe de Recherche en Transplantation & Immunologie du Sang de Cordon (GRETISC), Centre Cancérologie Charles-Bruneau, Centre de recherche du CHU Sainte-Justine, Montréal, Québec, Canada; ^2^ Département de Microbiologie Infectiologie & Immunologie, Université de Montréal, Québec, Canada; ^3^ Département de Pédiatrie, Université de Montréal, Québec, Canada; ^4^ Département de Sciences Biomédicales, Université de Montréal, Québec, Canada

**Keywords:** pediatric acute lymphoblastic leukemia, natural killer cells, plasmacytoid dendritic cells, TRAIL, interferon-alpha

## Abstract

Acute lymphoblastic leukemia (ALL) still frequently recurs after hematopoietic stem cell transplantation (HSCT), underscoring the need to improve the graft-versus-leukemia (GvL) effect. Natural killer (NK) cells reconstitute in the first months following HSCT when leukemia burden is at its lowest, but ALL cells have been shown to be resistant to NK cell-mediated killing. We show here that this resistance is overcome by NK cell stimulation with TLR-9-activated plasmacytoid dendritic cells (pDCs). NK cell priming with activated pDCs resulted in TRAIL and CD69 up-regulation on NK cells and IFN-γ production. NK cell activation was dependent on IFN-α produced by pDCs, but was not reproduced by IFN-α alone. ALL killing was further enhanced by inhibition of KIR engagement. We showed that ALL lysis was mainly mediated by TRAIL engagement, while the release of cytolytic granules was involved when ALL expressed NK cell activating receptor ligands. Finally, adoptive transfers of activated-pDCs in ALL-bearing humanized mice delayed the leukemia onset and cure 30% of mice. Our data therefore demonstrate that TLR-9 activated pDCs are a powerful tool to overcome ALL resistance to NK cell-mediated killing and to reinforce the GvL effect of HSCT. These results open new therapeutic avenues to prevent relapse in children with ALL.

## INTRODUCTION

Acute lymphoblastic leukemia (ALL) is the most common childhood cancer. Despite progress in chemotherapy and hematopoietic stem cell transplantation (HSCT), ALL remains a leading cause of death by cancer in children [1]. The cure rate of children with early relapse is only 50%, even after aggressive chemotherapy followed by allogeneic HSCT, highlighting the need for new therapeutic strategies [2, 3]. One of the most promising avenues of research is the increase of the graft-versus-leukemia (GvL) effect post-HSCT by harnessing the donor-derived immune system to eradicate leukemia.

Natural killer (NK) cells are the first lymphocytes to recover following allogeneic HSCT, when the residual leukemic burden is at its lowest [4–6]. As innate cytotoxic lymphocytes able to recognize and eliminate infected or tumor cells without prior sensitization, NK cells play a major role in the GvL effect post-HSCT [7]. Tumor cell killing by NK cells requires activating signals triggered by NK cell-activating receptors that recognize tumor-associated stress-induced molecules expressed by tumor cells [8–10]. NK cells express several activating receptors including the C-type lectin NKG2D, the DNAX accessory molecule-1 (DNAM-1) and natural cytotoxicity receptors (NCRs) such as NKp30, NKp44 and NKp46. NK cell cytotoxic activity can be further enhanced by cytokine stimulation, such as type I interferons (IFN) or by interactions with activated dendritic cells [11].

Activating signals integrated by NK cells are counterbalanced by inhibiting signals conferred by the engagement of NK cell inhibitory receptors, such as killer immunoglobulin-like receptors (KIR) and the heterodimer NKG2A/CD94, through the recognition of various class I human leukocyte antigens (HLA class I) expressed by target cells [9, 12]. In order to induce NK cell-mediated lysis, inhibitory signals must be repressed (by HLA downregulation or KIR/HLA mismatch) or activating signals must be increased through NK cell activation by cytokines, dendritic cells and the engagement of activating receptors [9, 11]. The cytolytic pathways engaged by NK cells to kill target cells include the polarized release of cytotoxic granules and the induction of apoptosis via ligands of the tumor necrosis factor (TNF) family such as FAS-L and the TNF-related apoptosis-inducing ligand (TRAIL) [13–15]. TRAIL and FAS-L induce apoptosis through the cross-linking of their respective death receptors, DR4 (TRAIL-R1) DR5 (TRAIL-R2) and FAS, on target cells.

While the role of NK cells in the GvL effect against acute myeloid leukemia (AML) has been well established, *in vitro* as well as clinical data showed that ALL blasts were more resistant to NK cell-mediated lysis. This is due not only to high levels of HLA class I expression, but also to low levels of stress-inducible proteins such as the ligands of the NKG2D receptor (MHC class I-related chains A and B – MICA/B and the members of the UL16-binding protein family), as well as low levels of adhesion molecules such as LFA-1 [16–18]. However, as recent studies provided evidence that activating signals can overcome NK cell inhibition by KIR ligands [19, 20], we explored new ways to activate NK cells in order to overcome ALL resistance to NK cell-mediated lysis.

Plasmacytoid dendritic cells (pDCs) are an attractive therapeutic tool to increase the cytolytic activity of NK cells [21]. Upon stimulation of their Toll-like receptors (TLRs), pDCs produce high amounts of type I IFNs, as well as several cytokines and chemokines that act on NK cells to increase their lytic activity [22, 23]. Recent reports have provided evidence that pDCs initiate and coordinate specific anti-tumor responses for which NK cell cytotoxic activity is required [24, 25]. Moreover, their direct interactions with NK cells has been shown to trigger NK cell cytotoxic activity against NK cell-resistant malignancies [22].

In this study, we used three pre-B ALL cell lines that differed in their levels of expression of NK cell activating ligands and HLA molecules. All of these cell lines were resistant to NK cell-mediated lysis in the absence of prior NK cell stimulation. We hypothesize that activation of NK cells by TLR-9 activated pDCs could overcome ALL resistance. We also explored the activating pathways involved in NK cell activation by TLR-9 activated pDCs as well as the cytolytic pathways involved in ALL lysis.

## RESULTS

### NK cell stimulation by TLR9-activated pDCs overcomes the resistance of ALL cells to NK cell killing

**Figure 1:**
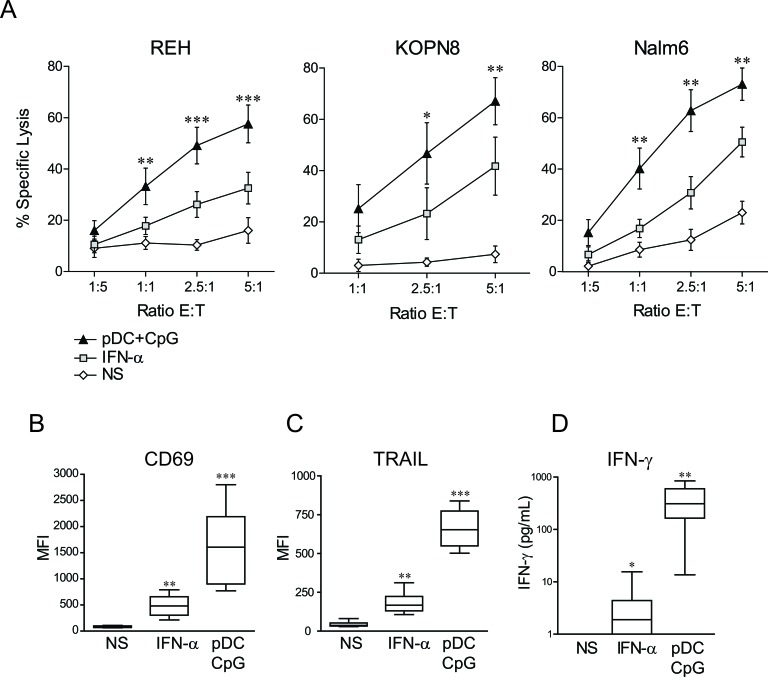
NK cell stimulation by TLR-9 activated pDCs overcomes ALL resistance to NK cell-mediated lysis and induces a unique activated phenotype **A.** Purified human NK cells were stimulated overnight with IFN-α (1000 IU/mL), co-cultured with activated pDC (pDC+CpG) or unstimulated (NS). Cytotoxic assays were then performed against REH, KOPN8 and Nalm6 cells. Means of n experiments performed in triplicates (*n* = 5 for REH, *n* = 3 for Nalm6 and KOPN8) are shown. Means of specific lysis ± SEM are represented for each ALL cell line and indicated E:T ratio. Paired t-test was used for statistical comparison **B.**-**C.** Activated NK cell phenotype was assessed by flow cytometry and **D.** IFN-γ production by ELISA following overnight stimulation with IFN-α or co-culture with TLR9-activated pDCs. Box plots represent the distribution of MFI for CD69 and TRAIL immune-stainings or IFN-γ concentrations (first and third quartiles, median and standard error bars) (*n* = 9 independent experiments with different donors of NK cells and pDCs). Friedman's test with Dunn's post-test was used for statistical comparison. ***p ≤ 0.001, **p ≤ 0.01, *p ≤ 0.05.

We tested whether NK cell stimulation by activated pDCs could enhance NK cell lytic functions against pre-B ALL. We assessed the susceptibility of three pre-B pediatric ALL cell lines to NK cell-mediated lysis, including KOPN8 cell line harboring the MLL translocation t(11;19). Human NK cells were isolated from adult volunteer's peripheral blood samples, while pDCs were either freshly isolated from PBMC or *in vitro* differentiated from cord blood-CD34^+^ progenitors. Cytotoxic assays revealed that overnight stimulation of NK cells by pDCs significantly increased NK cell cytotoxic activity against all three pre-B ALL cell lines tested (Figure 1A). ALL specific lysis reached 60-80% at an E:T ratio of 5:1, depending on the target cell line. No significant differences were observed in NK cell activation depending on the pDC source (Supplemental Figure S1). Accordingly, we have previously showed that *in vitro* differentiated pDCs produce large amounts of IFN-α upon TLR stimulation and display the same phenotype as mature peripheral blood pDCs [26]. A direct TLR-9 stimulation of NK cells by CpG ODN was ruled out, as CpG ODN alone did not increase NK cell cytotoxic activity against pre-B ALL cell lines (Supplemental Figure S2A). Moreover, unstimulated pDCs failed to enhance NK cell lytic activity, indicating that TLR-9 engagement on pDCs was required to enhance NK cell cytolytic functions (Supplemental Figure S2A). The lytic activity of TLR9-activated pDCs was also tested and, in the absence of NK cells, activated pDCs failed to induce pre-B ALL lysis, despite their high surface expression of TRAIL (Supplemental Figures S2B and S2C).

### High expression levels of TRAIL on NK cells stimulated by activated pDCs

The phenotype of activated NK cells following overnight co-culture with activated pDCs was then assessed. A marked upregulation of the CD69 activation marker on pDC-stimulated NK cells (Figure 1B) was observed. The expression of TRAIL was also highly increased on NK cells following stimulation with activated pDCs (Figure 1C). In contrast, the expression of several other molecules remained unchanged, including FAS-L, the NK cell activating receptors DNAM-1, NKG2D, NKp30, NKp44 and NKp46 and the intracellular levels of perforin and granzyme B (Supplementary Figure S3). NK cell stimulation by activated pDCs thus led to the expression of membrane molecules on NK cells.

### High IFN-γ production in response to NK cell stimulation with activated pDCs

As IFN-γ production is another hallmark of NK cell activation, IFN-γ levels were measured following NK cell stimulation by activated pDCs. IFN-γ production was significantly increased when NK cells were co-cultured with TLR-9-activated pDCs (Figure 1D), showing that activated pDCs induced not only NK cell cytolytic activity against ALL but also cytokine production.

### NK cell activation by TLR-9-activated pDCs is contact independent and IFN-α dependent

**Figure 2:**
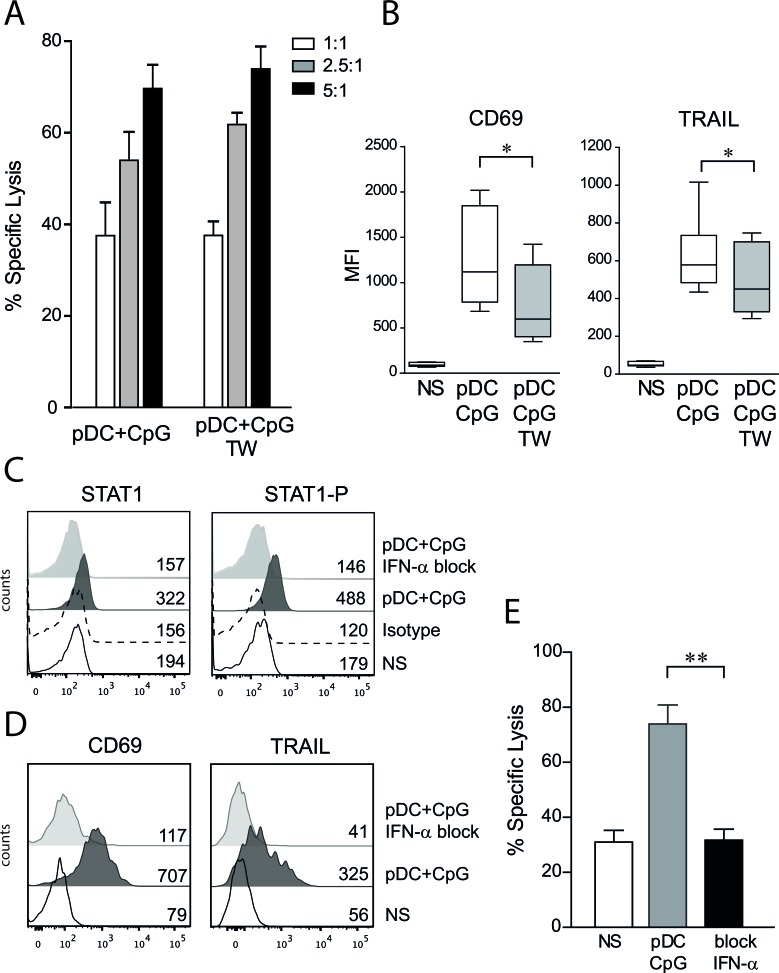
NK cell activation by TLR9-stimulated pDCs is contact independent and IFN-α dependent **A.** Human NK cells were co-cultured overnight with activated pDCs in contact or separated with a semipermeable membrane (transwell, TW). No difference in NK cell cytolytic functions was observed in cytotoxic assays performed against REH cells at indicated E:T ratio. The percentage of specific lysis (means of 3 independent experiments ± SEM) is presented. **B.** The expression levels of CD69 and TRAIL were assessed by flow cytometry. The MFI means ± SEM are presented (*n* = 9), paired t-test was used for statistical comparison *p ≤ 0.05. **C.** Adding anti-IFN-α and anti-IFN-receptor antibodies during NK/pDCs co-culture inhibits the type I IFN pathway as assessed by intracellular staining of phosphorylated-STAT1 and STAT1. Histograms are shown with MFI for unstimulated NK cells (NS), isotype control and NK cells co-cultured with activated pDCs with or without specific blocking antibodies (*n* = 3). **D.** CD69 and TRAIL upregulation was abolished in presence of blocking anti-IFN-α antibodies. Histograms are shown with MFI (*n* = 3). **E.** Blockade of type I IFN pathway abrogates pDC-induced anti-leukemic activity of NK cells as assessed by cytotoxic assays (*n* = 4, E:T=5:1). Paired t-test was used for statistical comparison **p ≤ 0.01.

Whether cell contact between activated pDCs and NK cells was required for enhancing NK cell lytic functions was then investigated. TLR-9 activated pDCs were co-cultured in contact with NK cells or separated by a semi-permeable porous membrane in transwell plates allowing for the diffusion of soluble factors. We observed that cytotoxic activity was similar for NK cells co-cultured with activated pDCs in contact or in transwell systems (Figure 2A). However, CD69 and TRAIL expression levels were lower on NK cells stimulated by activated pDCs in the absence of cell contact (Figure 2B). A combination of blocking monoclonal antibodies against IFN-α and the type I IFN receptor was then used. The blocking of the IFN-α pathway was first confirmed by intracellular staining of STAT1 and the phosphorylated form of STAT1 (Figure 2C). We observed that CD69 and TRAIL up-regulation on NK cells was entirely dependent on IFN-α production by activated pDCs (Figure 2D). The anti-leukemic activity of NK cells also was totally abrogated in the presence of blocking antibodies against the IFN-α pathway (Figure 2E). Although these results demonstrate that signaling through the type I IFN pathway is required to overcome pre-B ALL resistance to NK cell-mediated lysis, IFN-α stimulation alone was unable to reproduce the NK cell activation profile and NK cell lytic activity observed with co-culture with activated pDCs. Indeed, when used at a dosage corresponding to the amount of IFN-α produced by pDCs [27], IFN-α induced lower TRAIL and CD69 expression levels, lower IFN-γ production and lower lytic activity against ALL cells than with NK cells stimulated with activated pDCs (Figure 1).

### KIR/KIR-L blockade further enhances NK cell cytotoxicity against pre-B ALL

**Figure 3:**
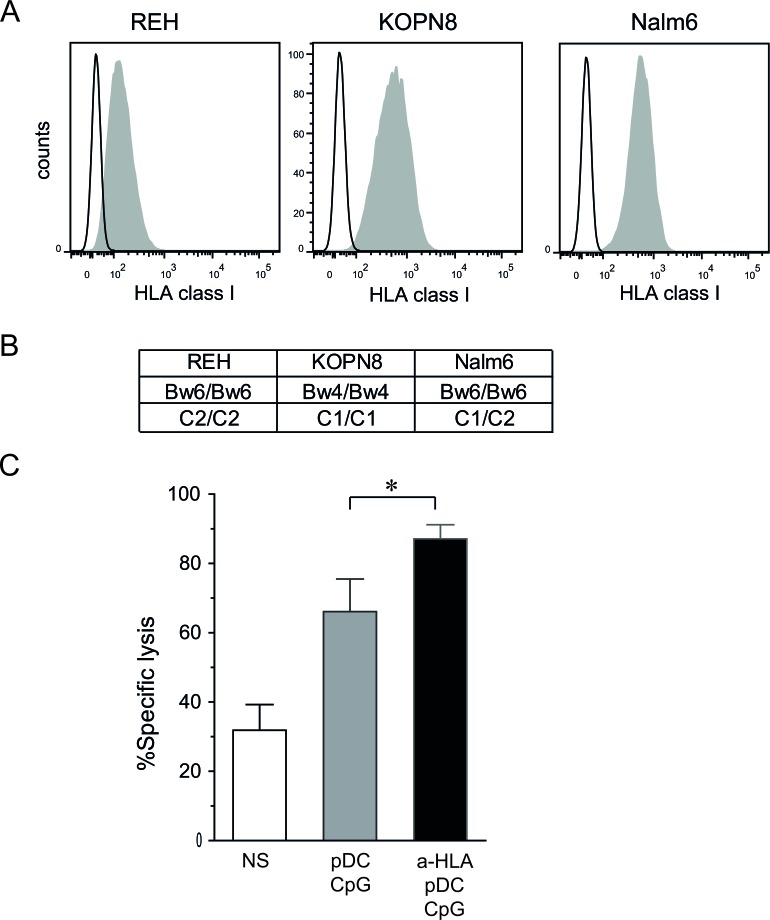
KIR/KIR-L blockade enhances NK cell cytotoxicity against pre-B ALL **A.** Pre-B ALL cell lines express various levels of HLA class I molecules as assessed by flow cytometry. Representative histograms are shown. **B.** HLA class I genotype is shown for REH, KOPN8 and Nalm6 cells. **C.** Cytotoxic assays against Nalm6 (5:1 E:T ratio) were performed with KIR/HLA class I matched NK cells (Bw6 C1/C2 donors) in the presence or absence of HLA blocking antibodies. Means of 3 independent experiments performed in triplicates are shown. Means of specific lysis are represented with SEM. Paired t-test was used for statistical comparison *p ≤ 0.05.

As shown in Figure 3A and 3B, the three pre-B ALL cell lines used exhibit various genotypes of HLA class I and various levels of HLA class I molecules at their surface. To test whether inhibiting KIR/KIR-L interactions increases NK cell cytotoxicity, Nalm6 cells were chosen, since they expressed significant levels of HLA class I molecules, and we were able to find several matched NK cell donors (i.e. C1/C2 Bw6 genotype). However, mismatched donors could not be found due to the C1/C2 genotype of this cell line. The addition of blocking antibodies against HLA class I molecules significantly increased the cytotoxic activity of pDC-activated NK cells as assessed by cytotoxic assays (Figure 3C). This result indicates that the enhancement of ALL killing by NK cells activated with pDCs can be further enhanced by a KIR-HLA mismatch between donor and recipient in the HSCT setting.

### TLR9-activated pDCs enhance NK cell degranulation against ALL

**Figure 4:**
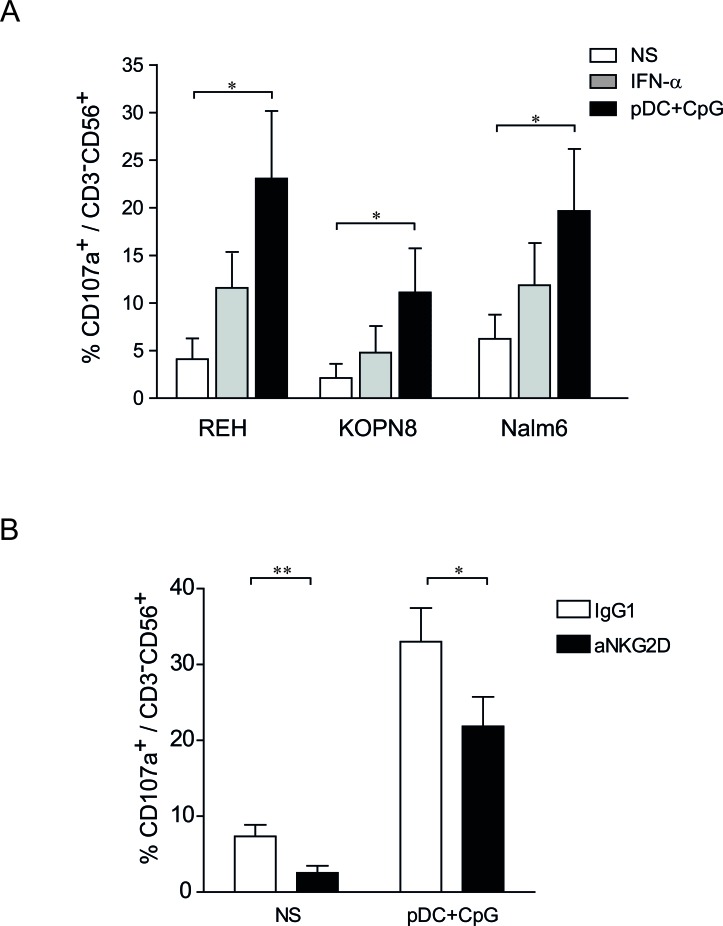
NK degranulation against ALL is increased following stimulation by TLR9-activated pDCs **A.** Purified human NK cells were stimulated overnight with IFN-α (1000 IU/mL), co-cultured with activated pDC or unstimulated. NK cells were then incubated with REH, KOPN8 and Nalm6 target cells at ratio 1:1 for 4 h and CD107a expression was assessed by flow cytometry. Graphs represent means ± SEM of the percentages of CD107a positive cells among CD56^+^CD3^−^ cells (*n* = 3). **B.** Unstimulated NK cells (NS) and pDC-stimulated NK cells were incubated with a blocking anti-NKG2D antibody or a control IgG1 before degranulation assay. NK cells were co-cultured with Nalm6 cells (ratio 1:1) for 4 h and the proportion of CD107a^+^ was assessed by flow cytometry. Percentages of CD107^+^ cells among CD3^−^CD56^+^ cells are presented with SEM (means of 4 independent experiments). Paired t-test was used for statistical comparison *p ≤ 0.05, **p ≤ 0.01.

NK cell degranulation was quantified by determining LAMP1 (Lysosomal-associated membrane protein-1)/CD107a surface expression on NK cells following effector:target contact.[28] We observed that unstimulated NK cells remained mainly negative for CD107a following incubation with ALL cells. However, the percentages of CD107a^+^ NK cells increased following NK cell activation. NK cells stimulated by activated pDCs exhibited a superior ability for degranulation than did IFN-α-stimulated NK cells (Figure 4A). Moreover, the proportion of CD107a^+^ NK cells was higher upon contact with REH and Nalm6 cells than with KOPN8 cells, indicating that factors present on target cells determine the capacity of NK cell degranulation.

### NKG2D, but not DNAM-1, plays a role in the killing of ligand-positive ALL cells

**Figure 5:**
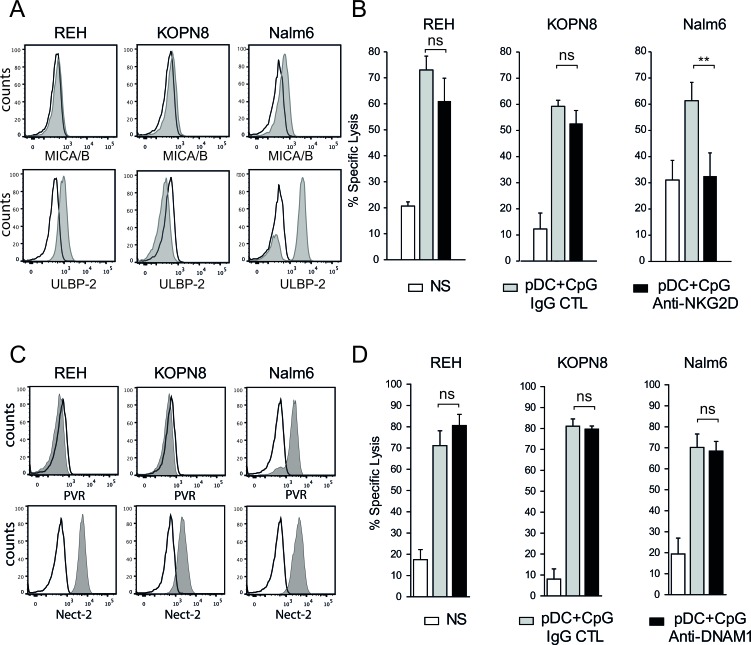
NKG2D, but not DNAM1, plays a role in pre-B ALL lysis by activated NK cells **A.** The expression of the NKG2D ligands, MICA/B and ULBP2 was assessed by flow cytometry. Histograms are shown for each pre-B ALL cell line (black line: isotype control; gray filled specific antibody). **B.** The addition of blocking anti-NKG2D antibody decreased NK cell cytolytic activity against Nalm6 but not against REH and KOPN8 cells (5:1 E:T ratio). Specific lysis means of 3 independent experiments performed in triplicates are represented ± SEM. Paired t-test was used for statistical comparison, **p ≤ 0.01 **C.** The expression of the DNAM1 ligands, Nectin-2 and PVR, was assessed by flow cytometry (black line: isotype control; gray filled specific antibody). Representative histograms are shown for each pre-B ALL cell line. **D.** The addition of blocking anti-DNAM1 antibody did not affect NK cell cytolytic activity against all 3 pre-B ALL cell lines (5:1 E:T ratio). Specific lysis means of 3 independent experiments performed in triplicates ± SEM are represented.

The NK cell activating receptors engaged by ALL cells to induce NK cell degranulation and cytolytic functions were then determined. We confirmed that pre-B ALLs expressed low levels of NKG2D ligands (MICA/B and ULBP-2), except for Nalm6 cells that expressed high levels of ULBP-2 (Figure 5A) [17, 29]. Accordingly, blocking NKG2D resulted in a decrease in Nalm6 specific lysis (Figure 5B) and a concomitant decrease in CD107^+^ expression on activated NK cells (Figure 4C). Although ALL cell lines expressed ligands for the DNAM1 receptor, i.e., Nectin-2 for all three cell lines and PVR for Nalm6 (Figure 5C), the addition of a blocking antibody against DNAM1 did not reduce the cytotoxic activity of activated NK cells against any of the three ALL cell lines (Figure 5D). The anti-DNAM1 antibody was, however, able to specifically inhibit NK cell cytotoxicity against other cancer cell lines (data not shown). Collectively, these results indicate that the NKG2D-mediated release of cytotoxic granules plays a role in NK cell lytic activity against some pre-B ALL cells, depending on the presence of the corresponding activating ligands. However, DNAM-1 does not play any role even in the presence of its ligands on ALL cells.

### TRAIL-mediated apoptosis plays a major role in pre-B ALL killing by activated NK cells

**Figure 6:**
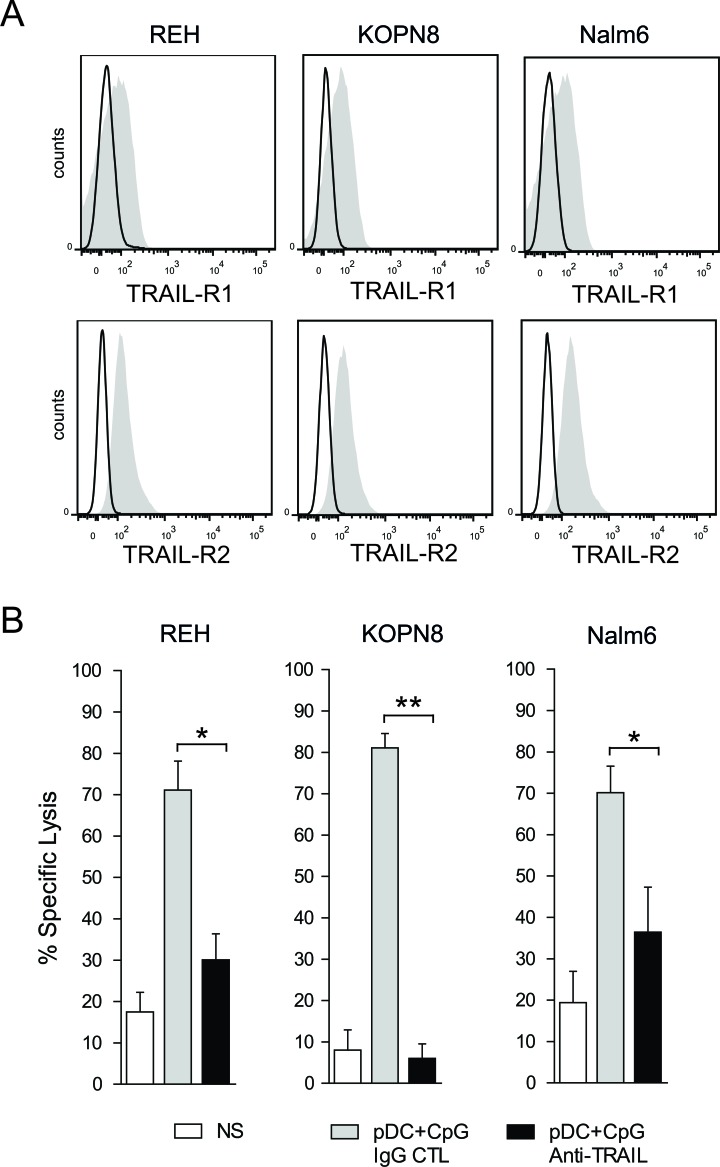
TRAIL mediated apoptosis plays a major role in pre-B ALL lysis by activated NK cells **A.** All 3 pre-B ALL cell lines express the death receptors, TRAIL-R1 and TRAIL-R2, as assessed by flow cytometry. Representative histograms are shown for each pre-B ALL cell line (black line: isotype control; gray filled specific antibody). **B.** NK cell-mediated cytotoxic activity against all 3 pre-B ALL is significantly inhibited by the blockade of TRAIL/TRAIL-R interactions, using a specific blocking antibody against TRAIL (5:1 E:T ratio). Paired t-test was used for statistical comparison, **p ≤ 0.01, *p ≤ 0.05.

Since we observed that activated pDCs induced a high expression of TRAIL on activated NK cells, the role of TRAIL-mediated apoptosis in the killing of ALL by pDC-activated NK cells was investigated. We first observed that all three ALL cell lines expressed the death receptors TRAIL-R1 (DR4) and TRAIL-R2 (DR5) (Figure 6A). We further showed that the specific lysis of pre-B ALL was inhibited in the presence of a blocking antibody against TRAIL. The lysis of KOPN8 was completely abrogated by TRAIL blockade, whereas lysis inhibition for REH and Nalm6 was 66% and 50 % respectively (Figure 6B). These results show that the NK cell-mediated cytotoxicity of some ALL cell lines, such as KOPN8, is completely dependent on TRAIL induced apoptosis, whereas TRAIL plays a major role in the killing of other cell lines, such as REH and Nalm6.

### Activated pDCs control human pre-B ALL in humanized mice

**Figure 7:**
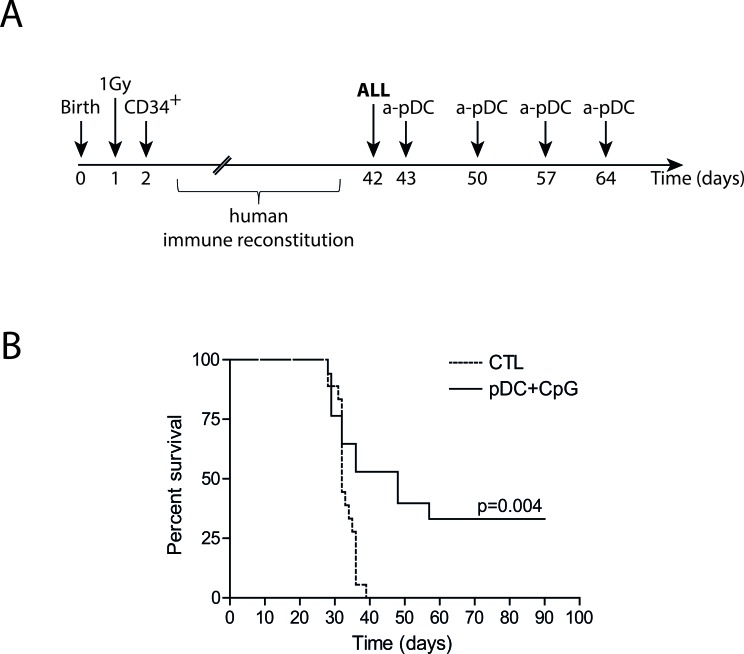
Activated pDC infusions delay leukemia onset in humanized mice **A.** Experimental design. Irradiated newborn NSG mice were transplanted with 10^4^ human cord blood-derived CD34^+^ cells. 10^5^ REH ALL cells were intravenously injected in humanized mice 5-6 weeks after transplantation, followed by four weekly injections of activated pDCs or saline solution for control mice (CTL). **B.** Survival curves of ALL-bearing humanized mice treated with activated pDCs or saline solution injections. Mice were sacrificed after overt leukemia onset. Flow cytometry analysis of bone marrow samples confirmed complete leukemia involvement. Log-rank test was used to compare survival.

We and others recently described that human CD34^+^ precursors can be cultured and differentiated in pDCs *in vitro* [26, 30]. This method allows the production of high numbers of functional pDCs that can be activated by TLR ligands. We verified that injection of TLR-9-activated pDCs in humanized mice induced *in vivo* NK cell activation (Supplemental Figure 4). Humanized mice bearing human ALL were treated by repeated injections of TLR-9-activated pDCs. As shown by Kaplan-Meier survival curves, the leukemia onset was significantly delayed and 30% of the mice survived (Figure 7). These results indicate that activated pDCs are a unique therapeutic tool to activate NK cells and to control ALL *in vivo*.

## DISCUSSION

We showed that the stimulation of NK cells by TLR-9-activated pDCs overcomes the resistance of ALL cells to NK cell-mediated killing. We further demonstrated that high expression of TRAIL was a hallmark of pDC-activated NK cells and that TRAIL-induced apoptosis of target cells is the major cytolytic pathway involved in ALL lysis by pDC-activated NK cells. The inhibition of KIR engagement by HLA class-I molecules further enhanced ALL lysis by activated NK cells. Finally, we provide evidence that NK cell stimulation by activated pDCs can control ALL *in vivo.*

Cross-talk between NK cells and dendritic cells (DC) is responsible for enhanced activation of both cell types and increases their antitumor activity [22, 31, 32]. Among DC subsets, pDCs are the major producers of type I IFN, an important activator of NK cell lytic functions [33]. Besides the CD69 up-regulation and IFN-γ production already reported by others,[34, 35] we showed in this study that activated pDCs induce a strong upregulation of TRAIL on NK cells. Accordingly, we found that pre-B ALL cell lysis by pDC-activated NK cells is inhibited by the blockade of TRAIL/death receptor interactions. Given that a majority of pre-B ALL blasts express TRAIL-R1 and/or TRAIL-R2 [36] and that pre-B leukemia-initiating cells are sensitive to TRAIL-mediated apoptosis [37], our findings are of particular clinical interest to prevent ALL-relapse following HSCT. Our data, though, indicate that the release of cytotoxic granules by pDC-activated NK cells does not play a major role in pDC-induced NK cell cytotoxicity against ALL. The low expression of NK cell receptor ligands by ALL or the absence of co-activating signal required to triggered NK cell degranulation [38] may be responsible for the minor role of NK cell release of cytolytic granules. Future studies will delineate precisely the mechanisms of degranulation in the lysis of ALL cells by NK cells stimulated by activated pDCs.

Overcoming the resistance of pre-B ALL to NK cell-mediated lysis opens new therapeutic opportunities for post-transplant NK cell-based immunotherapy in children with high-risk ALL. Indeed, NK cells are the first lymphocytes to recover after HSCT during the first two months post-transplant, when leukemia burden is at its lowest after the radio-chemotherapeutic conditioning regimen [4–6]. Increasing the anti-leukemia function of donor-derived NK cells could therefore decrease the relapse rate and improve the survival of children with ALL. The production of IFN-γ by pDCs-activated NK cells is also expected to be beneficial in the post-HSCT context. Indeed, it has been shown in several models that IFN-γ enhances the GvL effect and also protects against GvHD [39]. Our *in vitro* and *in vivo* data pave the way for future post-transplant NK cell stimulation by adoptive transfer of third-party activated pDCs. Sufficient amounts of pDCs for adoptive transfer cannot be obtained from peripheral blood because adult blood contains very low numbers of pDCs [40]. Fortunately, we and others have described methods for *in vitro* expansion and differentiation of human pDCs from cord blood progenitors. Indeed, up to 10^7^ functional human pDCs are obtained from cord blood units, making adoptive transfers of activated pDCs clinically feasible [26, 30, 41].

In agreement with our results, TLR-9 agonist administration has been shown to induce anti-leukemia responses in mouse models [42, 43]. However, in the clinical HSCT setting, the therapeutic efficacy of TLR-9 ligands will depend on the presence of TLR-9 responsive pDCs. Studies have shown that pDC reconstitution is delayed following bone marrow transplantation, reaching normal blood pDC counts one year after transplantation [44, 45]. We recently reported that despite the rapid reconstitution of pDC counts after cord blood transplantation, these pDCs display impaired IFN-α production in response to TLR-9 stimulation [26]. A low number or impaired function of pDCs during the first months after HSCT is thus a limitation for the use of TLR-9 agonists in this setting. Fortunately, our *in vivo* data show the feasibility of adoptive transfer of activated pDCs and their efficacy in controlling leukemia. Further pre-clinical experiments are currently underway to increase the efficacy of this approach.

pDCs have advantages over other candidates for NK cell activation after HSCT such as cytokine stimulation with IL-2, IL-15 or IFN-α. The systemic administration of inflammatory cytokines such as IL-2 induces severe side effects related to T lymphocyte proliferation and activation, i.e., severe graft-versus-host disease (GvHD) or proliferation of regulatory T cells that decreases the GvL effect [46, 47]. IL-15 has recently been reported as a promising therapeutic cytokine to stimulate both innate and adaptive immunity against tumors [48]. However, IL-15 also activates CD8^+^ cells, leading to a similar risk of GvHD. More importantly, a recent report has shown that IL-15 enhances pre-B ALL proliferation and dissemination in the central nervous system, precluding the use of this cytokine to increase NK cell functions in ALL patients [49]. Finally, our study shows that although the IFN-α pathway is absolutely required for pDC-induced NK cell activation, IFN-α alone is not able to reproduce TRAIL upregulation on NK cells and NK cell lytic activity against ALL observed with pDC-activated NK cells. This result suggests that other soluble factors participate in NK cell activation induced by activated pDCs. The characterization of these cytokines and chemokines is underway and will be of particular importance for future clinical applications.

Although several laboratory and clinical studies have shown that KIR mismatch alone is not sufficient to activate NK cells and eradicate ALL [50–54], we showed here that blockade of the HLA-KIR recognition further increases ALL lysis by pDC-activated NK cells. This finding has important clinical implications. NK cell stimulation by activated pDCs can be expected to be more efficient in the context of a KIR mismatch between donor and recipient. Similarly, it has recently been reported that the cytotoxicity of expanded NK cells against pediatric pre-B ALL was higher when donor NK cells were KIR mismatched with their targets [55]. Together, these results indicate that post-HSCT NK cell-based immunotherapy has a greater chance of success if donor derived NK cells are not inhibited by KIR/HLA interactions.

In conclusion, our data show that the resistance of ALL to NK cell lysis can be overcome by the use of activated pDCs to increase NK cell lytic functions. These data open new opportunities of post-transplant immunotherapy based on NK cell stimulation by activated pDCs with many advantages over other methods of post-transplant NK cell stimulation. Pre-clinical experiments are currently underway to refine this approach.

## MATERIALS AND METHODS

### ALL cell lines and cell culture

Three pediatric B-cell precursor ALL (pre-B ALL) cell lines resistant to NK cell-mediated lysis were used in this study: REH t(12;21), Nalm6 t(5;12) and KOPN-8 t(11;19) that harbors a rearrangement of the MLL gene (DSMZ Braunschweig, Germany). Cell lines were maintained in RPMI-1640 (Wisent, Saint-Bruno, QC) medium supplemented with 10% heat-inactivated FBS (Wisent, Canada) and 1% Antibiotic-Antimycotic (Life Technologies, Burlington, ON) at 37°C in a 5% CO_2_ atmosphere. All cell lines were negative for mycoplasma contamination, as assessed by PCR using the Universal Mycoplasma Detection Kit (ATCC, Manassas, VA).

### pDC expansion and differentiation from cord blood CD34^+^ cells

Human pDCs were expanded and differentiated from purified cord blood-CD34^+^ progenitors as described [26]. Briefly, Cord blood units were obtained from the CHU Sainte-Justine Research Center cord blood bank with approval from the Institutional Review Board. Mononuclear cells were isolated by gradient centrifugation on Ficoll-PaquePLUS (GE Healthcare Bio-Science AB, Uppsala, Sweden). CD34^+^ progenitors were isolated by positive selection using CD34 antibody-conjugated magnetic microbeads and MiniMACS columns (Miltenyi Biotec, San Diego, CA) and were seeded at a density of 2 × 10^5^ cells/mL in serum-free expansion medium (StemSpan^TM^ SFEM, StemCell Technologies, Vancouver, BC). The cells were expanded for 7 days with recombinant human stem cell factor (10 ng/mL), thrombopoietin (50 ng/mL) and FLT3-ligand (100 ng/mL) (all from R&D System, Minneapolis, MN or Miltenyi Biotec). Expanded CD34^+^ hematopoietic stem cells (HSCs) were then cultured with rhIL-7 (10ng/mL, Cytheris, France), TPO and Flt3-ligand for 7 days of differentiation. The cultures were refreshed every 2 days and maintained for a total of 14 days at 37°C in a humidified incubator with 5% CO_2_. Cells were stained with the labelled mouse anti-human antibodies and pDCs (Lin^−^HLA-DR^+^CD123^+^) were sorted by flow cytometry (FACS Aria, BD Biosciences, San Jose, CA). The functional characteristics of expanded pDCs have been described elsewhere [26].

### NK cell and pDC isolation from peripheral blood

Peripheral blood mononuclear cells (PBMC) from healthy volunteers were isolated by Ficoll-PaquePLUS density gradient centrifugation. NK cells and pDCs were then isolated by negative selection with the EasySep^®^ NK Cell Enrichment Kit and the EasySep^®^ pDC Enrichment Kit (StemCell Technologies), respectively. The purity of NK cells and pDCs was always above 95%.

### NK cell stimulation

NK cells were plated in a 96-well round-bottom plate (2×10^6^ cells/mL) and incubated overnight without stimulation or with human IFN-α (1000 UI/ml) or co-cultured with pDCs (Lin^−^HLA-DR^+^CD123^+^) (ratio NK:pDC of 10:1) and CpG-A ODN2216 (10μg/ml) (Invivogen, San Diego, CA) at 37°C in a 5% CO_2_ atmosphere. Of note, pDCs and NK cells were not prepared from the same donor. In some experiments, 24-well microplates equipped with a transwell insert (0.4μm, Greiner bio-one) were used to prevent direct contact between NK cells and pDCs. For IFN-α signaling neutralization assays, NK cells were incubated for 30 minutes with neutralizing anti-IFN-α/β receptor Chain2 and anti-IFN-α antibodies (20μg/ml each) (MMHAR-2 and MMHA-2 respectively; PBL Assay Science, Piscataway, NJ), prior to the addition of IFN-α or pDCs and overnight incubation.

### Cytotoxic activity assays

The ability of NK cells to kill pre-B ALL cell lines was assessed by cytotoxic assays using flow cytometry with the LIVE/DEAD^®^ Cell-mediated cytotoxicity kit (Life Technologies, Invitrogen). Target cells were labelled with the green-fluorescent membrane dye DiOC_18_. NK cells were harvested after overnight stimulation. NK and target cells were then plated in triplicates at different Effector:Target (E:T) ratios (ranging from 1:5 to 5:1) in a 96-well coned plate. Cells were briefly spun to provide immediate contact between NK cells and targets and then incubated for two hours at 37°C. Dead cells were then stained with propidium iodide (PI) and counting beads were added. Flow cytometry analysis was performed using an LSR Fortessa cytometer (BD Bioscience, San Jose, CA) and data were analyzed with FlowJo software (Tree Star, Ashland, OR). Absolute numbers of live target cells were assessed (DiOC^+^PI^−^) and data were expressed as the percentage of specific lysis calculated by the following formula: specific lysis (%) = [(#absolute live cells –; #experimental live cells)/(#absolute live cells)] × 100. For blocking experiments, after Fc receptor saturation with purified mouse IgG1, NK cells were incubated with 30μg/ml of purified NA/LE mouse anti-human CD253 (TRAIL) mAb or purified NA/LE mouse anti-human CD226 (DNAM-1) mAb (BD Pharmingen, San Jose, CA) or anti-human NKG2D (Amgen, Mississauga, ON) for 30 minutes at RT as previously described [56].

### Immunophenotypic analysis by flow cytometry

NK cell phenotype was assessed by flow cytometry after overnight stimulation with IFN-α or TLR9-activated pDCs. Unstimulated NK cells were used as controls. All conjugated monoclonal antibodies were purchased from BD Biosciences or Biolegend. We used FITC-conjugated anti-CD69 and anti-Granzyme B; PE-conjugated anti-NKp44, anti-Fas-ligand, anti-perforin, anti-NKG2D, anti-DNAM-1 and anti-TRAIL; alexa-fluor® 647-conjugated anti-NKp30; brilliant violet 421™-conjugated anti-NKp46. Granzyme B and perforin protein expression were assessed by intracellular staining as previously described [28]. The expression of death receptors, NKG2D and DNAM-1 ligands as well as HLA class I molecules was assessed on pre-B ALL cell lines using APC-conjugated anti-DR4, PE-conjugated anti-DR5, PE-conjugated anti-PVR, PE-conjugated anti-Nectin2, PE-conjugated anti-MICA/MICB, PE-conjugated anti-ULBP2 and PE-conjugated anti-HLA class I. Flow cytometry analysis was performed with LSR Fortessa cytometer and data were analyzed with FlowJo software.

### Dosage of IFN-γ production

NK cells were stimulated overnight as described above. Supernatants were collected and stored at −80°C. IFN-γ dosage was performed by enzyme-linked immunosorbent assay (ELISA) (PBL InterferonSource, Piscataway, NJ).

### Degranulation assays

CD107a staining was used to assess NK cell degranulation against ALL cell lines as previously described [28]. Briefly, after an overnight incubation with activated pDCs or IFN-α, NK cells were harvested and cultured with ALL cells at 1:1 E:T ratio, in the presence of PE-conjugated CD107a- specific mAb (BD Biosciences). Monensin (Golgi-stop, BD Biosciences) was added to the co-culture one hour after the beginning of the incubation to avoid the degradation of reinternalized proteins from the surface. Cells were incubated for three additional hours and then stained with APC-conjugated anti-CD56 and PE-Cy7-conjugated anti-CD3 before being analyzed by flow cytometry.

### *In vivo* control of leukemia

*Nod*/Scid/*IL-2Rγ*^−/−^ mice were purchased from the Jackson Laboratory and maintained in pathogen-free conditions. Humanized mice were generated as previously described [57]. Briefly, newborn (1-3-day-old) mice received sub-lethal (1 Gy) total body irradiation from an X-ray source (Faxitron CP160), and were injected intra-hepatically with 10^4^ human CD34^+^ purified from cord blood units. Protocols for generating humanized mice were approved by our local Animal Care Committee according to the guidelines of the Canadian Council on Animal Care in Science. To promote human NK cell differentiation, humanized mice received human IL-15/IL-15Rα-Fc complex (ALT803, Altor Biosciences [58]) once a week for seven weeks starting six weeks after transplantation. To reproduce human ALL in humanized mice, 10^4^ REH cells were injected intravenously 8 weeks after transplantation, followed 24 h later by infusions of activated pDCs (10^5^ pDCs per mouse). pDC injections were repeated once a week for five weeks. A group of humanized mice injected with REH cells and treated with saline injection was used as control. Leukemia development was monitored by weekly bleeding and mice were sacrificed when overt leukemia signs were reached.

### Statistics

All *in vitro* experiments were repeated at least three times with different blood donors for NK cell and pDC purification and all results are presented as the mean of n experiments to eliminate donor-derived variation. Animal experiments were performed three times with five mice in each group; the survival curve presented includes all the 15 mice in treated and untreated groups. Animals were allocated into groups after NK cell numbering to ensure comparable NK cell reconstitution among groups. Animal experiments were not blinded. For *in vitro* experiments, we used the two-sided paired t-test for statistical analysis of paired data and, when multiple comparisons were required, we used Friedman's test with Dunn's post-test. *P* = 0.05 and 0.01 were chosen as levels of significance for paired t-tests and multiple comparisons, respectively. Log-rank test was used to compare survival curves.

## SUPPLEMENTARY MATERIAL FIGURES


