# Supplementary material for: TRAIL-mediated killing of acute lymphoblastic leukemia by plasmacytoid dendritic cell-activated natural killer cells
Source: Oncotarget. 2015 Jul 22;6(30):29440–55. doi: 10.18632/oncotarget.4984 (PMC4745738; doi:10.18632/oncotarget.4984)
Supplement: Supplementary file 1 [file oncotarget-06-29440-s001.pdf]

The importance of microenvironment: the role of CCL8 in metastasis formation of melanoma

Supplementary Material

Supplementary Figure S1

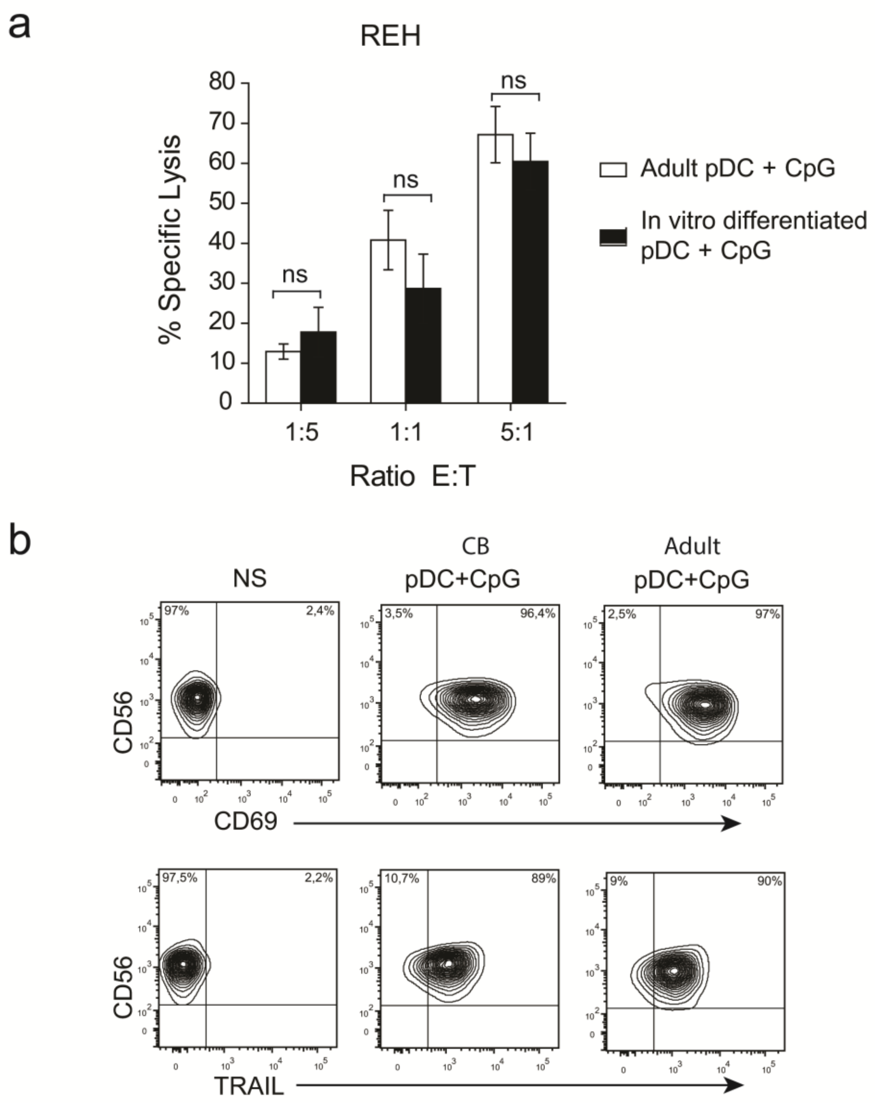

NK cells stimulated by peripheral blood pDCs (from adult volunteers) or by *in vitro* differentiated pDCs (from $CD34^+$ cord blood cells) exhibit similar cytotoxic activity against pre-B ALL. (**a**) Cytotoxic assays were performed against REH cell line. Peripheral blood NK cells and pDCs were isolated from adult volunteers while *in vitro* differentiated pDCs were sorted from culture of $CD34^+$ cells. Means of specific lysis are reprensented with SEM (n=3-8). (**b**) Phenotypic analysis of NK cells co-cultured with cord blood derived-pDCs or adult peripheral blood pDCs. Similar up-regulation of CD69 and TRAIL was observed. Representative contour plots are presented (n=8).

Supplementary Figure S2

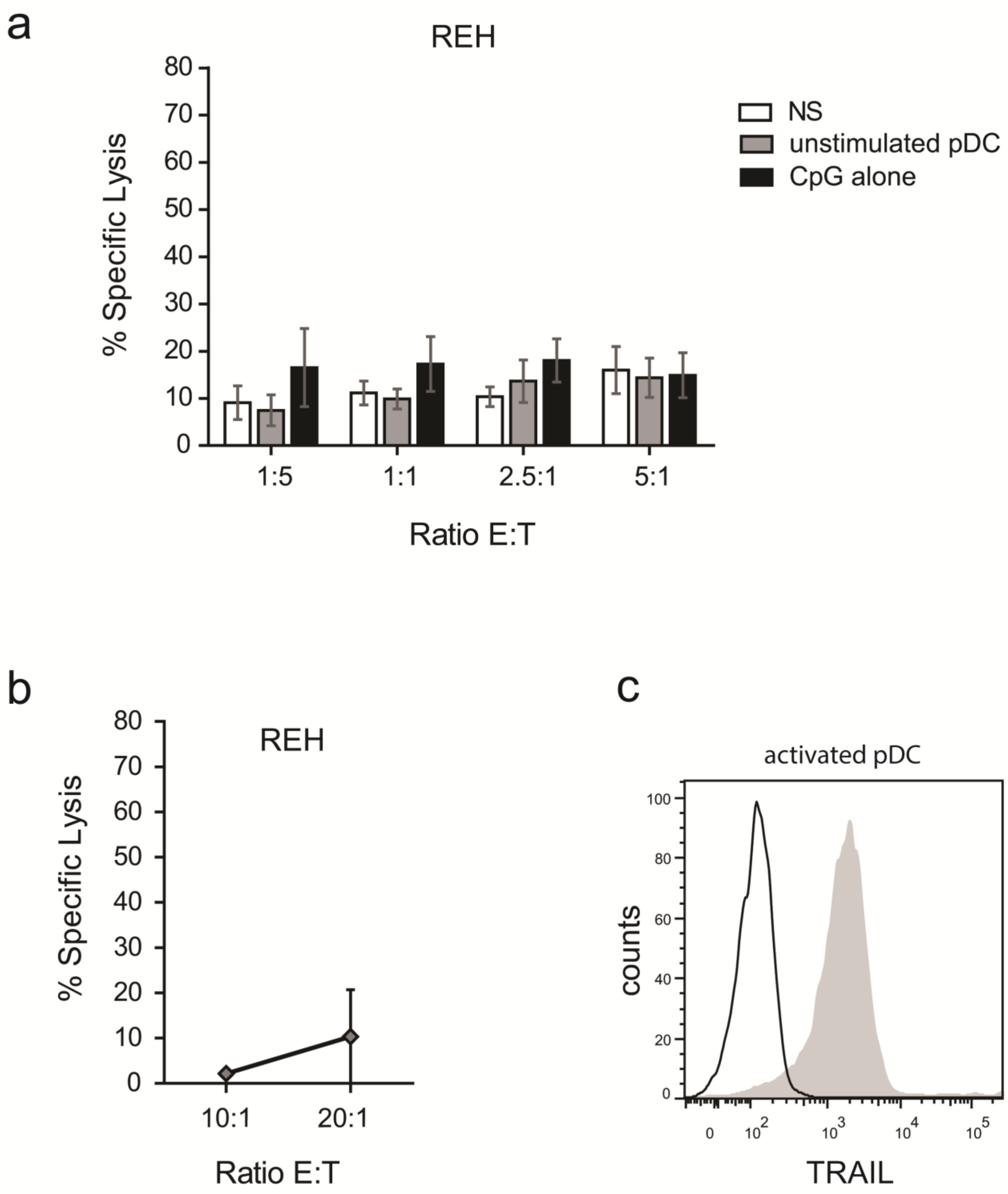

N**K cells stimulated with CpG alone, co-cultured with unstimulated pDC are unable to kill pre-B ALL**. (**a**) Means of specific lysis of REH cells with SEM are represented (n=3). (**b**) Cytotox assays were performed against REH cells with activated pDCs as effectors. Means of specific lysis triplicates are represented with error bars (n=1). (**c**) TRAIL surface expression on TLR-9 activated pDCs was assessed by flow cytometry. Histograms are shown for unstimulated pDCs (black line) and activated pDCs (gray fill) (n=3).

Supplementary Figure S3

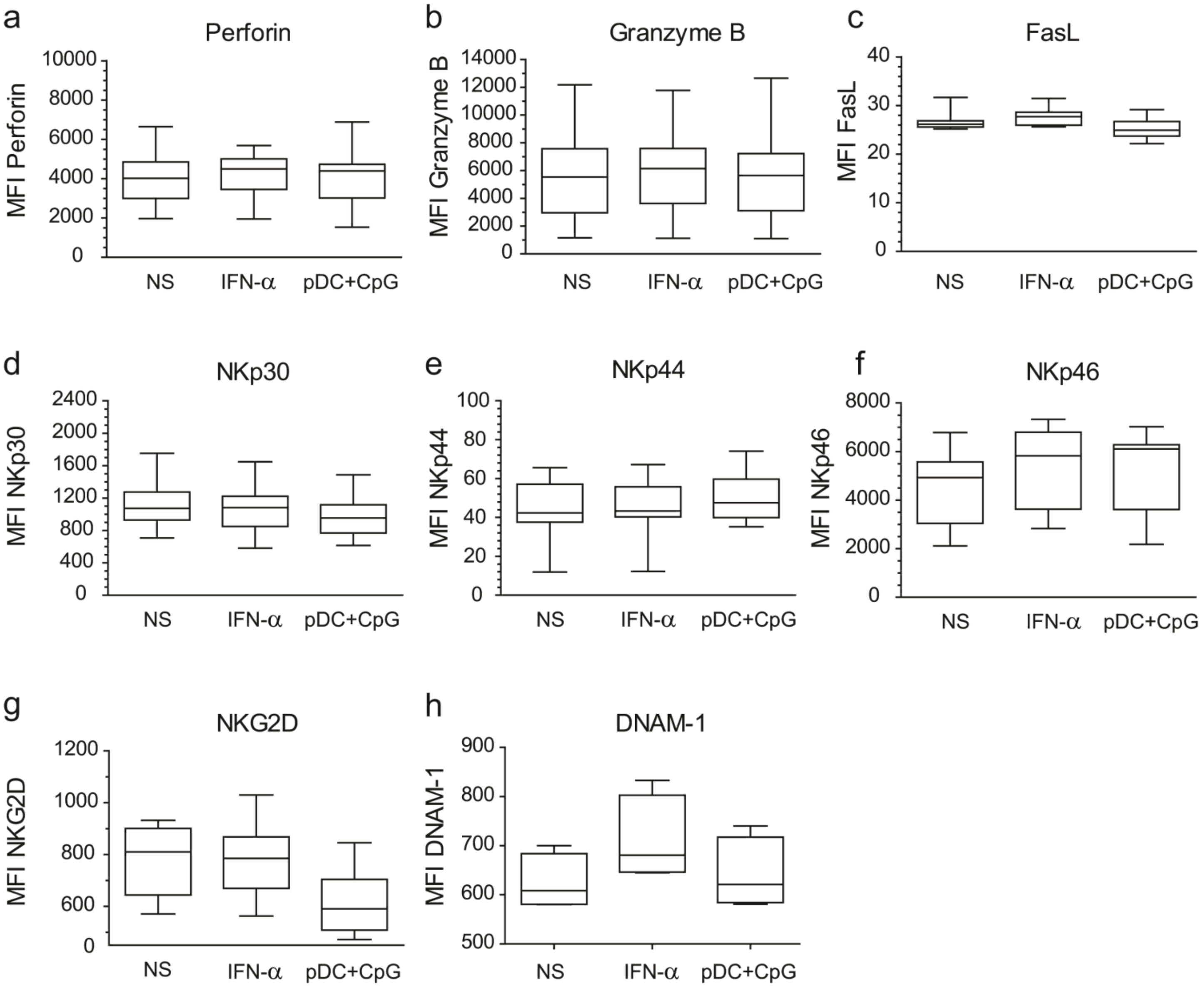

Phenotype analysis of activated NK cells.
Following overnight stimulation with either IFN-α (1000IU/mL) or co-culture with TLR9-activated pDC, NK cells were stained with specific indicated antibodies. The means of fluorescence intensity (MFI) are showed for each marker (n=4-14 independent experiments), data are presented as box plots showing the median of each distribution (horizontal bar), the 25th and 75th percentile (box) and the range (vertical bar).

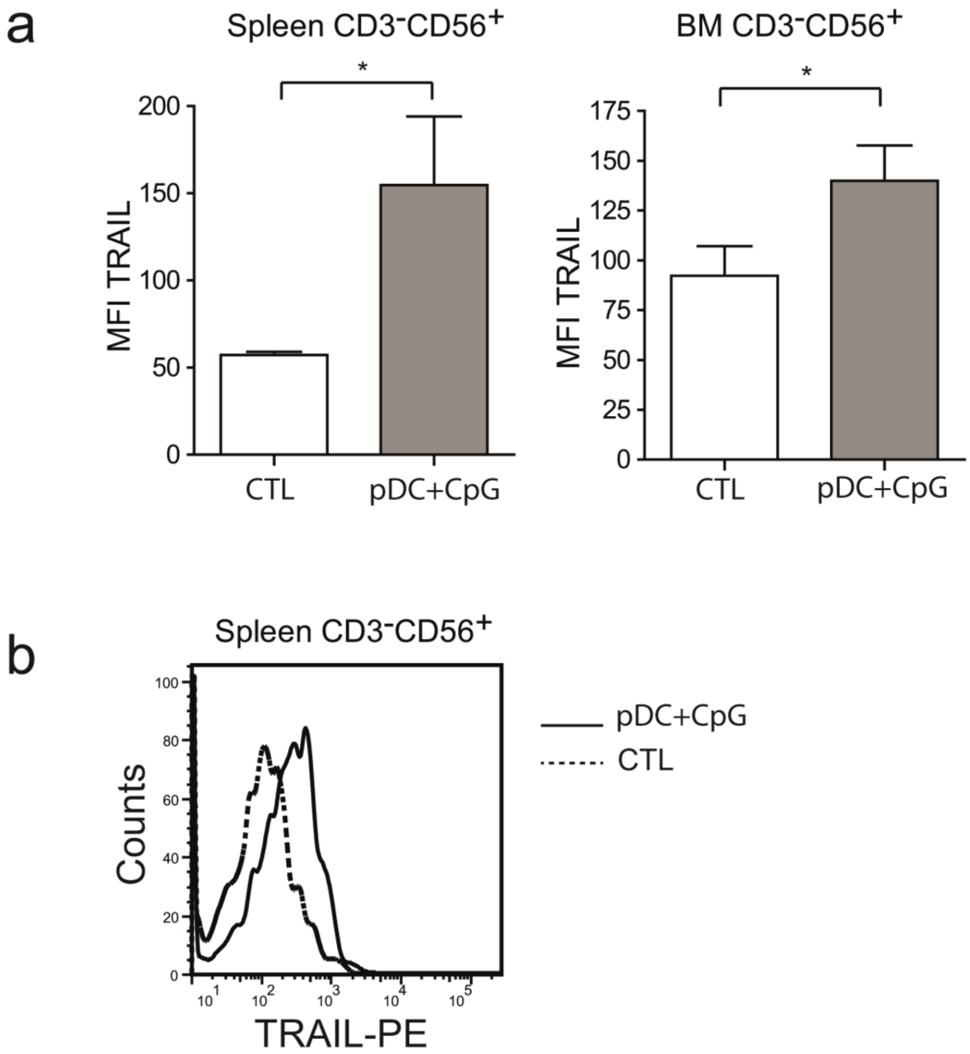

Phenotype analysis of *in vivo* activated NK cells.
TLR-9 activated pDCs (10^5 cells per mouse) or saline solution (CTL) were intravenously injected in humainized mice. Twenty four hours later, mice were sacrificed, bone marrow (BM) and spleen were havested and analysed by flow cytometry. (**a**) The expression of TRAIL was significantly increased on spleen and BM NK cells from mice injected with activated pDCs as compared with control mice. The graphs represent the means of median fluorescence intensity (MFI) with SD (n=6 mice in pDC+CpG group and n=4 mice in CTL group). * p=0.04 (**b**) A representative histogram is presented showing increased TRAIL expression on spleen NK cells after injection of activated pDCs.
